# The relationship between brain behavioral systems and the characteristics of the five factor model of personality with aggression among Iranian students

**DOI:** 10.5249/jivr.v8i2.696

**Published:** 2016-07

**Authors:** Saeid Komasi, Mozhgan Saeidi, Ali Soroush, Ali Zakiei

**Affiliations:** ^a^Clinical Research Development Center, Imam Reza Hospital, Kermanshah University of Medical Sciences, Kermanshah, Iran.; ^*b*^Cardiac Rehabilitation Center, Imam Ali Hospital, Kermanshah University of Medical Sciences. Kermanshah, Iran.; ^*c*^Lifestyle Modification Research Center, Imam Reza Hospital, Kermanshah University of Medical Sciences, Kermanshah, Iran.; ^*d*^Social Development and Health Promotion Research Center, Kermanshah University of Medical Sciences, Kermanshah, Iran.

## Abstract

**Background:**

Aggression is one of the negative components of emotion and it is usually considered to be the outcome of the activity of the Behavioral Inhibition and the Behavioral Activation System (BIS/BAS): components which can be considered as predisposing factors for personality differences. Therefore, the purpose of this study was to investigate the relationship between brain behavioral systems and the characteristics of the five factor model of personality with aggression among students.

**Methods:**

The present study has a correlation descriptive design. The research population included all of the Razi University students in the academic year of 2012-2013. The sampling was carried out with a random stratified method and 360 people (308 female and 52 male) were studied according to a table of Morgan. The study instruments were Buss and Perry Aggression Questionnaire, NEO Personality Inventory (Short Form), and Carver and White scale for BAS/BIS. Finally, SPSS20 was utilized to analyze the data using Pearson correlation, regression analysis, and canonical correlation.

**Results:**

The data showed a significant positive relationship between the neurosis and agreeableness personality factors with aggression; but there is a significant negative relationship between the extroversion, openness, and conscientiousness personality factors with aggression. Furthermore, there is a significant positive relationship between all the components of brain behavioral systems (impulsivity, novelty seeking, sensitivity, tender) and aggression. The results of regression analysis indicated the personality characteristics and the brain behavioral systems which can predict 29 percent of the changes to aggression, simultaneously.

**Conclusions:**

According to a predictable level of aggressiveness by the personality characteristics and brain behavioral systems, it is possible to identify the personality characteristics and template patterns of brain behavioral systems for the students which be presented to them as a necessary training in order to control and manage of anger and aggression.

## Introduction

Aggression can be defined as an instinctive behavior that depending on the circumstances can be divided into two forms of proportional and disproportional. The proportional aggression is an adaptive behavior that like the social values can be changed along with changing communities; but disproportional aggression is considered to be inefficient and non-adaptive behavior when faced with perceived or real risk.^1^ A harmful behavior is considered as an aggression if it is done deliberately, in order to hurt one’s self or others. Aggression may lead to detrimental effects, such as damaging, harmful attacks to themselves or others, sudden death, and risk behaviors. ^2^ Typically, expressing and controlling anger is conceptualized in terms of four major components: exporting or external expression of the anger, including the anger toward other persons or objects found in the environment; the internal expression of anger which refers to the orientation of this anger toward one's own mind or its suppression. Controlling export or expressing anger that is defined by avoiding to express it toward other people or around objects. Finally, inhibition of import or internal anger that is related to the control and suppression of emotions through relaxation or being calm upon being angry.^3^

Theories of aggression and personality argue that personality factors are the key predictors for aggressive behavior^4^ and the results of various studies ^5-10^ indicate that there may be certain personality traits influencing the occurrence or non-occurrence of aggressive behavior. Barthelme, ^11^ during the study of the relationship between aggression and the salient five personality dimensions, realized that there is a significant relationship between the five dimensions of personality and aggression. Furthermore, the results of the reviews of Shirvani and Mahdipour ^12^ show that there is a significant positive correlation between aggression and some personality characteristics such as neurosis. The personality, which refers to the relatively stable pattern for individual states and behaviors represent an individual’s desires, influenced by internal factors such as thoughts, values, hereditary traits, and the external factors such as observable behaviors. ^13^ According to the theory of Pavlov, personality is based on the performance of the neural system and the excitation and inhibition which are considered as two fundamental processes governing the overall activity. If the internal and the external stimuli which are causing the excitation and inhibition, are repeated, they will become more stable in the brain and they will ultimately create dynamic behaviors which are strong. ^14^ Then, Aysenk proposed a two-dimensional model of personality and motivation on the basis of neuropsychology, and tried to introduce a structure and a biological function for each of the properties by talking about traits like extroversion, introversion, neuroticism and psychosis as the main factors of personality. ^15^ However, Gray suggested another theory in this area in the year 2000.

Gray suggested three different brain-behavioral systems on the theory of Reinforcement Sensitivity Theory (RST) that are predisposing for personality differences: the Behavioral Inhibition System (BIS), the Behavioral Activation System (BAS), and the War-escape System.^16^ He attributed the individuals’ responses to the environmental stimuli to both the behavioral inhibition and activation system^17^ that are rated based on neurology: BAS is responsible for tendency behaviors in response to reward (positive affect) and BIS is responsible for the inhibitory behaviors in response to threats and punishment (negative affect). In other words, BAS makes up the person to be sensitive in the case of the potential rewards and finds a motivation for searching the rewards. BIS can affect a person's sensitivity towards the punishment and makes up the person to be sensitive in the case of the potential punishment by avoiding it.^18^ According to the researchers, one of the components of the negative affect is the emotion of anger and aggression and which is thought to be anger as the consequence of the activity of BAS and BIS.^19^ On the other hand, the innate tendency to experience negative affect such as aggression about self and others can be a risk to a person's health by destroying it gradually.^20^ Now, according to the latest studies and theories about the possible role of brain behavioral systems^19,21^ and the five factor model of personality and the characteristics on the anger and aggressive behaviors, the aim of this study was to investigate the relationship between brain behavioral systems and the characteristics of the five-factor model of personality with aggression among students.

## Methods

**Study Design**

This study has a correlation descriptive design. The research population included all of the Kermanshah Razi University students studying in the bachelor and master's degrees, in the academic year 2012-2013.

**Inclusion Criteria**

Inclusion criteria comprised of (1) not having an academic probation being in the age range 18-32 years, and (2) the lack of employment to study in other time period.

**Sampling and Data Collection**

Then, sampling was carried out with a random stratified method. In this case, 400 people, from seven faculties belonging to the Razi University, were selected based on the students in each faculty and according to the table of Morgan. Finally, the data were analyzed for the 360 people (308 female and 52 male). From among the participants, 40 of them were set aside as they did not have the inclusion criteria of the study or their questionnaires were not readable. The data collection was carried out with the use of Buss and Perry Aggression Questionnaire, NEO Personality Inventory (Short Form), and Carver and White scale for BAS/BIS.

**Instruments**

The Buss and Perry Aggression Questionnaire has 29 questions and the measurements physical aggression, verbal aggression, anger, and hostility. Based on normalization in Iran, test-retest reliability for the all test and subtests have been reported 0.80 and a Cronbach's Alpha of 0.76 was obtained for the angry subtest.^22^

NEO Personality Inventory (Short Form) has 60 questions which evaluates the five personality factors: neurosis, agreeableness, extroversion, openness, and conscientiousness. Each factor covers 12 questions a score between 0-48 is assigned to each factor (each question: 0-4 score). The long form questionnaire has 240 questions and is produced by McCrae and Costa for normal population. The reported alpha coefficients were 0.74 to 0.89 with the average of 0.81. By the same token, a recent study about personality and eating disorders has reported the internal consistency of 0.69-0.90 for the scales of the test.^23^ Also, Haghshenas has confirmed the reliability of this test in Iran through the implementation of the test on a sample of 502 people in Shiraz, using both test-retest and Cronbach's alpha.^24^

Carver and White scales for BAS/BIS were prepared by Carver and White.^25^ This scale consists of 24 self-report questions and included three subscales: drive, response to reward, searching for entertainment. Of course, four additional options have been brought in the scale as covering items which do not have a role in the assessment of the BAS/BIS. The scoring procedure is based on a four-point Likert scale ranging from absolutely correct to absolutely incorrect. The question of the scale are grading reversely, except for questions 2 and 22. According to the report of Carver and White, ^25^ the internal consistency for BIS subscale is 0.74 and the internal consistency for three subscales: drive, response to reward, searching for entertainment are 0.73, 0.76, and 0.66, respectively. Furthermore, the internal consistency for BIS subscale has been 0.87 and for BAS subscales has been 0.82, 0.77, and 0.86, in a study by Abdollahi et al.^26^

**Statistical analysis**

Finally, Data of 360 people were analyzed. The mean and standard deviation of continuous variables were reported. The data were evaluated in terms of the normal distribution using the Kolmogorov-Smirnov test and it was clear that the distribution is normal; That is, p values for the variables were greater than 0.05. The SPSS Software for Windows (ver. 20.0) was utilized to analyze the data using Pearson correlation, regression analysis, and canonical correlation.

## Results

Of 360 participants, 308 were female (85.6%) and 52 were male (14.4%). Mean (SD) age in total was 23.3(±4.3), 22.9 (±3.7) in females group, and 24.2 (±4.9) in males group. The mean and standard deviation of the evaluated variables are shown in Table 1.

**Table 1:** The mean and the standard deviation of the evaluated variables.

Variables	Minimum	Maximum	Mean	Standard deviation
Neuroticism	15	57	36.05	7.71
Extroversion	14	45	30.22	6.43
Agreeableness	20	45	33.58	4.55
Openness	18	46	31.27	5.26
Conscientiousness	14	45	28.31	7.21
Impulsivity	4	16	9.61	2.63
Novelty seeking	4	16	7.80	2.30
Tender	5	15	7.21	2.24
Sensitivity	9	23	14.97	2.79
Aggression	43	76	59.71	7.18

The correlation coefficients between the personality characteristics and brain behavioral systems with aggression are shown in Table 2.

**Table 2:** The correlation coefficients between the personality characteristics and brain behavioral systems with aggression.

Predictor variables	Aggression									
	Physical		Verbal		Anger		Hostility		Total score	
	r	P	r	P	r	P	r	P	r	P
**Personality Characteristics**										
Neuroticism	0.09	0.06	0.33	0.001	0.26	0.001	0.24	0.001	0.32	0.001
Extroversion	0.03	0.48	-0.07	0.18	-0.01	0.74	-0.24	0.001	-0.12	0.02
Agreeableness	0.14	0.006	0.07	0.18	0.13	0.01	0.02	0.67	0.10	0.04
Openness	-0.22	0.001	-0.30	0.001	-0.15	0.004	-0.32	0.001	-0.38	0.001
Conscientiousness	-0.08	0.11	-0.22	0.001	-0.15	0.005	-0.28	0.001	-0.26	0.001
**Brain Behavioral Systems**										
Impulsivity	0.16	0.001	0.30	0.001	0.25	0.001	0.11	0.03	0.30	0.001
Novelty seeking	0.32	0.001	0.22	0.001	0.14	0.006	0.11	0.03	0.28	0.001
Tender	0.24	0.001	0.07	0.13	0.03	0.53	0.03	0.47	0.12	0.01
Sensitivity	0.10	0.05	-0.01	0.75	0.16	0.002	0.16	0.002	0.15	0.004

**Fig.1:**
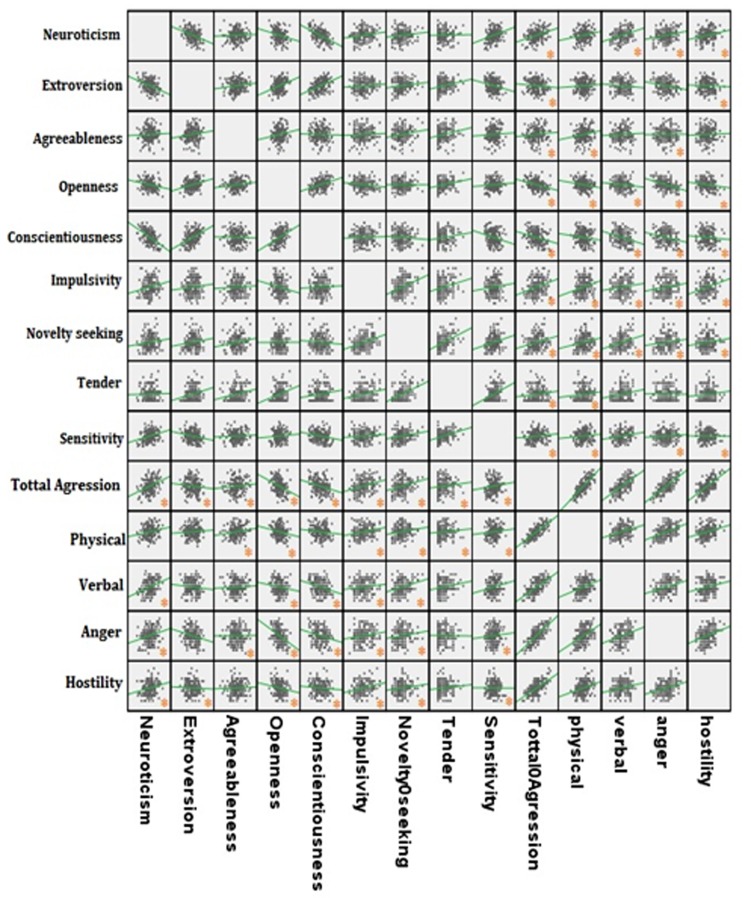
The correlation between the personality characteristics and brain behavioral systems with aggression components.

The results in Table 2 indicate that there is a correlation (r=0.32, P <0.001), between neurosis factor and total scores of aggression, between extroversion factor and aggression (r=-0.12, P <0.02), between agreeableness factor and aggression (r=0.10, P <0.04), between openness factor and aggression (r=-0.38, P <0.001), and between conscientiousness factor and aggression (r=-0.26, P <0.001). In addition, the data indicate that there is a significant correlation 0.30 and 0.28 (P <0.001) between impulsivity and novelty seeking subscales and total scores of aggression, respectively. Also, there is a significant correlation 0.12 (P <0.01) and 0.15 (P <0.004) between tender and sensitivity subscales and total scores of aggression, respectively (Fig.1).

The use of the step by step regression analysis for predicting aggression based on the personality characteristics and the brain behavioral systems can be seen in Table 3.

**Table 3:** predicting aggression based on personality characteristics and brain behavioral systems.

Criterion variable	Summary of the model	Predictive variable	B	β	t	Significance level
	R= 0.54	Openness	-0.46	-0.33	7.14	0.001
	R2= 0.29	Novelty seeking	0.59	0.19	3.92	0.001
aggression	F= 29.73	Neurosis	0.17	0.18	3.89	0.001
	P<0.001	Impulsivity	0.37	0.13	2.81	0.005
		Agreeableness	0.18	0.11	2.54	0.01

The results of regression analysis indicate that the predictive model is significant at p<0.001 which is done in five steps. Accordingly, the fifth step results show both the behavioral characteristics and the brain systems which are able to predict 29% of the changes related to aggression. Also, openness, neurosis, and agreeableness personality characteristics can predict aggression with the beta coefficients -0.33, -0.18, and 0.11, respectively, and impulsivity and seeking novelty can predict aggression with the beta coefficients of 0.19 and 0.13, respectively. 

Table 4 shows the results of the canonical correlation coefficient between the personality subscales and the brain behavioral systems with dimensions of aggression.

**Table 4:** Standard coefficients, structure coefficients, and other parameters of the canonical correlation analysis.

Variables	Coefficients		
	Standard coefficients	Structure coefficients	Shared variance
Neurosis	0.40	0.66	
Extroversion	0.006	-0.24	
Agreeableness	0.17	0.17	
Openness	-0.55	-0.67	Wilkes lambda = 0.48
Conscientiousness	-0.13	-0.52	RS= 0.52
Impulsivity	0.26	0.55	F= 7.63
Seeking novelty	0.19	0.45	P < 0.001
Tender	0.18	0.17	
Sensitivity	-0.05	0.18	

The parameters of correlation analysis in Table 4 show that among the predictor variables, weight the openness subscale (-0.55) is most associated with the first combination or fundamental variables derived from the dependent variables (components of aggression). The structural coefficient for openness subscale is -0.67. The extraversion subscales (with standard ratio -0.006) has a minimal role in predicting aggression. Wilks lambday test level is 0.48; In other words, about 52 percent of the variance of the dependent variables are predicted (components of aggression).

## Discussion

The present study examined the relationship between brain behavioral systems and the characteristics of the five factor model of personality with aggression among students. The results showed that there was a significant positive relationship between the neurosis and agreeableness personality factors with aggression; but there was a significant negative relationship between the extroversion, openness, and conscientiousness personality factors with aggression. This finding is consistent with the findings of some studies.^5-9,11,12,27-30^

To explain these findings, especially the existence of the positive relationship between the neurosis and agreeableness personality factors with aggression, it can be said that people inability to resist the impulses and temptations and violent behaviors are regarded as a signs of high levels of neurosis.^12^ Furthermore, sometimes much flexibility can lead to aggressive behaviors. Persons higher in agreeableness may display more negative affect than other persons because receiving negative feedback may represent a greater mismatch of their interpersonal orientation. The persons may be more sensitive to the damaging effects of destructive interpersonal tactics and therefore express more anger in interactions that use these destructive tactics. ^30^ But to explain the three components of extroversion, openness, and conscientiousness, that are associated with aggression negatively, it can be said that people with low scores on extroversion are non-social and inactive who usually do not want to discuss and express their emotions properly. ^31^ The people without openness do not accept the ideas of moral and social characteristics and have fewer health and behavioral stability. Also, the people without conscientiousness are non-purposeful, irregular, less conscientious and more unsuccessful in controlling their behavior who suffer from lack of energy. ^12^ Thus, it can be said that those who have less control over their behavior are more likely to show aggression. Also, they showed poor disposition to experience fear, a constellation of deficient empathy, disdain for and lack of close attachments with others, which can be lead to high proactive aggression.^29^

Furthermore, the results of the study showed that there was a significant positive relationship between all the components of the brain behavioral systems (impulsivity, novelty seeking, sensitivity, and tender) and aggression. The finding are in line with the findings of some studies.^19,21,25,27,32^ In order to explain these data, and based on the approach of neuropsychology in the field of study of the structure of the brain, it can be said that BIS which contains the septo-hippocampal system -and its monoaminergic sensory neurons radiate of the brain stem and the neocortical regions in the frontal segment provide the basis of motivational for behavioral inhibition that may lead to negative consequences, especially on pulsing damage and new tissues. ^33^ So, BIS is able to set up the physical processes and the interaction of higher cortical and have a role on the cognitive and affective responses to the environmental challenges.^15^ Thus, the existence of a significant positive relationship between BIS and negative affection is predictable; and since anger is considered to be one of the components of negative affection, there is a justified relationship between this component and the BIS.^21^

On the other hand, the existence of a positive relationship between anger (that is considered to be one of the components of negative affection) and the BAS can be justified due to the relationship between the increase activity between the left frontal cortex and the BAS. Harmon-Jones & Sigelman,^32^ found that the relative activity of left frontal cortex - that has a clear correlation with BAS- has a relationship with negative emotions of anger. By the same token, the results of two surveys showed there is a relationship between increasing the activity of left frontal cortex and the decreasing activity of the right frontal cortex with trait anger ^34^ and state anger.^32^

It should be noted that in this study the population was limited to students and the results cannot be generalized to other groups. It is recommended that similar studies should be done among other segments of society such as other groups who are more involved in the everyday interactions.

## Conclusion

Our findings showed that there is a significant positive relationship between the neurosis and agreeableness personality factors with aggression; but there is a significant negative relationship between the extroversion, openness, and conscientiousness personality factors with aggression. Furthermore, there are significant positive relationships between all the components of Brain Behavioral Systems (impulsivity, novelty seeking, sensitivity, and tender) and aggression. According to a predictable level of aggressiveness by the personality characteristics and the Brain Behavioral Systems we can identify the personality characteristics and template patterns of brain behavioral systems for the students which be presented to them as a necessary training to control and manage anger and aggression.

## Acknowledgments

It is hereby deemed necessary to thank and appreciate the staff of Clinical Research Development Center of the Imam Reza Hospital, Cardiac Rehabilitation Unit of the Imam Ali Hospital, and Social Development and Health Promotion Research Center of the Kermanshah University of Medical Sciences for their cooperation in providing data.
